# A review of occupational noise-induced hearing loss: focus on mechanisms and preventive measures

**DOI:** 10.1093/bmb/ldaf020

**Published:** 2025-11-25

**Authors:** Kow-Tong Chen, Shih-Bin Su

**Affiliations:** Department of Occupational Medicine, Tainan Municipal Hospital (Managed by Show Chwan Medical Care Corporation), Tainan 701, Taiwan; Department of Public Health, College of Medicine, National Cheng Kung University, Tainan 701, Taiwan; Department of Occupational Medicine, Chi-Mei Medical Center, Tainan 710, Taiwan

**Keywords:** occupational diseases, noise-induced hearing loss, epidemiology, pathogenesis

## Abstract

**Introduction:**

This research investigates the epidemiology, underlying mechanisms, and prevention strategies associated with occupational noise-induced hearing loss (ONIHL), while also presenting effective control measures.

**Source of data:**

Relevant literature was sourced from databases such as MEDLINE, PubMed, Embase, Web of Science, and Google Scholar, encompassing articles until February 2025.

**Areas of agreement:**

ONIHL is widely recognized as a common occupational health issue among workers. Additionally, noise can lead to psychological and physiological complications beyond direct auditory damage.

**Areas of controversy:**

The effects of noise differ across individuals, and the precise causes behind ONIHL remain poorly understood, although several pathways have been proposed.

**Growing points:**

New treatment options focused on oxidative stress, inflammation, and neuropathy are being developed through innovative drug delivery systems.

**Areas timely for developing research:**

There is a pressing need for molecular approaches to explore the mechanisms of ONIHL, particularly in the care of individuals with hearing disabilities.

## Introduction

Noise-induced hearing loss (NIHL) is a global public health concern. Occupational noise-induced hearing loss (ONIHL) refers to progressive sensorineural hearing loss resulting from prolonged exposure to occupational noise [1]. Historical records indicate that workers, such as copper miners in the 18th century, suffered hearing loss due to excessive noise exposure. According to the World Health Organization (WHO), ~10% of the global population is exposed to noise pollution, with 5.3% exhibiting NIHL [2]. Approximately 16% of cases of disabling hearing loss in adults are attributed to exposure to occupational noise. While ONIHL may not directly lead to early death, it significantly contributes to a high level of disability [1].

In the United States, ~25% of workers who report hearing difficulties attribute them to exposure to noise in the workplace [3]. Workers with hearing impairment face numerous challenges, including safety risks, increased absenteeism, and a higher likelihood of work-related injuries. The financial and health burdens of occupational noise exposure are substantial, leading to increased hospitalizations and higher healthcare costs [4]. Furthermore, ONIHL can severely impact an individual's communication abilities, leading to social stress, isolation, diminished self-esteem, and strained interpersonal relationships [1]. Research has linked mild hearing loss in older adults to a doubled risk of dementia, and significant hearing impairment is associated with a fivefold increase in risk. Noise exposure can trigger autonomic nervous system reactions and endocrine responses, potentially elevating the likelihood of developing hypertension, coronary artery disease, and stroke [2]. Identifying preventable risk factors is crucial, as timely diagnosis and intervention can help mitigate ONIHL. Understanding the mechanisms and distribution of ONIHL is vital for developing effective preventive measures, which is the focus of this review.

## Methods

**Figure 1:**
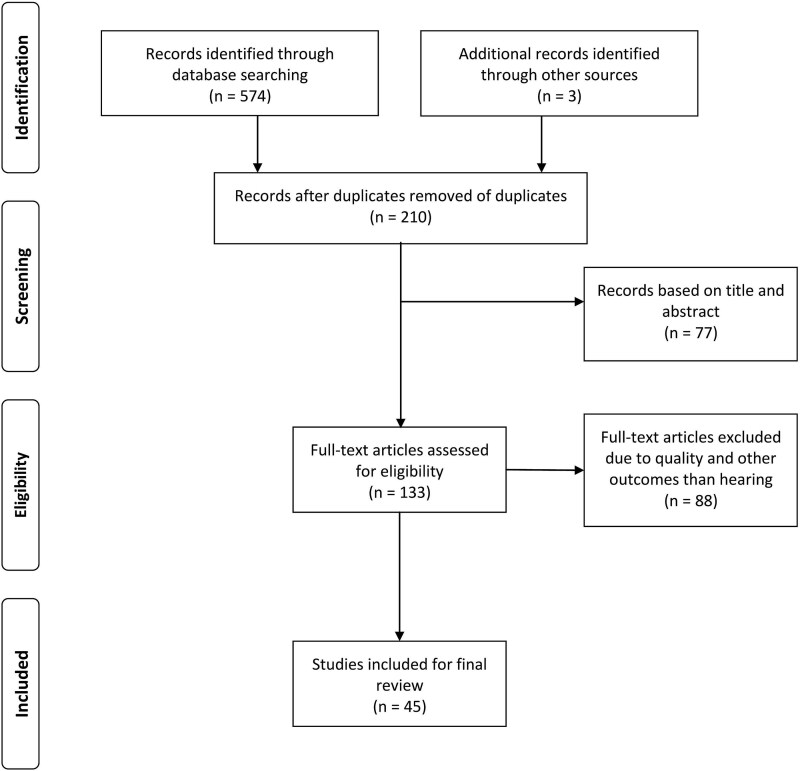
PRISMA flow diagram for the literature research.

This review examined multiple online databases, including MEDLINE (National Library of Medicine, Bethesda, Maryland, USA), PubMed, Embase, Web of Science, and Google Scholar. We utilized a combination of keywords, including “occupation,” “noise-induced hearing loss,” “epidemiology,” “pathogenesis,” and “protection,” to find pertinent published research. Additionally, we explored the references within the retrieved articles to uncover any additional relevant research. The review adhered to the Preferred Reporting Items for Systematic Reviews and Meta-Analyses (PRISMA) guidelines [5]. The articles included in this report were restricted to those published between January 2000 and February 2025. The criteria for including papers centered on studies that investigated exposure to occupational noise, either alone or in conjunction with other factors, and its association with hearing loss and various health-related issues. Our focus was on the research examining the relationships between workplace noise, hearing loss, and other related health issues. Conversely, our exclusion criteria comprised studies addressing chemical ototoxicity (such as exposure to toluene, carbon monoxide, or mercury), medications known to cause hearing loss (including aminoglycoside antibiotics and cisplatin), noise exposure from activities unrelated to occupational settings (e.g. lawn mowing, using power tools, train riding, attending concerts, significant sports events, and hunting), as well as cases involving a single instance of very high-level sound exposure resulting in acoustic trauma [1]. In our thorough methodology, we reviewed the reference lists of relevant studies to identify any publications that may have been overlooked. The literature review was completed in February 2025 (Fig. 1). We gathered relevant information on epidemiology, pathogenesis, and preventive strategies and organized it into appropriate categories.

## Defining occupational hearing loss and audiometric outcomes

ONIHL is characterized by partial or total hearing loss in one or both ears, resulting from noise exposure in the workplace, which usually progresses slowly over time [6]. There are two main types of hearing loss: temporary threshold shift (TTS) and permanent threshold shift (PTS) [7]. Acoustic trauma, resulting from a single exposure to an intense sound [8], was excluded from the definition of ONIHL in this study.

Experience the critical importance of pure tone audiometric testing—a key tool in identifying and measuring the extent of ONIHL [9]. This testing method relies on the active participation of individuals exposed to occupational noise, allowing for a subjective assessment of their hearing capabilities. Notably, ONIHL often presents itself through symmetrical noise exposures, characterized by distinct notches in high-frequency ranges—specifically at 3000, 4000, or 6000 Hz—recovering at 8000 Hz during audiometric evaluations [7]. These notches can manifest at specific frequencies without affecting adjacent ones. Yet, as exposure continues, the risk of developing notches at additional frequencies increases, mainly influenced by age-related hearing changes. Differentiating ONIHL from age-related hearing loss is crucial, especially in older individuals. Prolonged noise exposure can also lead to diminished hearing in lower frequencies (0.5, 1, or 2 kHz) [6]. Research indicates that ONIHL typically does not exceed 75 dB in high frequencies and 40 dB in lower frequencies. Still, alarming trends indicate that the rates of severe hearing loss are significantly higher among noise-exposed workers than in the general population [1]. While noise exposure can cause temporary shifts in audiometric thresholds, with recovery within 16 to 48 hours, repeated exposure can result in permanent changes. The WHO categorizes hearing impairment into four distinct grades—slight, moderate, severe, and profound—based on audiometric ISO values across various frequencies. Workplace assessments often combine self-reported hearing issues with physical examinations to effectively detect ONIHL. Typically, ONIHL presents symmetrically; however, atypical cases may arise due to uneven exposure levels between the ears. Research has shown that individuals may experience poorer hearing thresholds in one ear, even after accounting for factors like hand dominance [10].

Historically, the early 20th century marked a turning point in the ability to measure noise levels and hearing capabilities. Groundbreaking research has emphasized the distinctive notch at 4000 Hz, confirming that the intensity, frequency, and length of exposure are key factors in hearing loss [7]. Following World War II, efforts intensified to safeguard against NIHL, with the US Air Force laying down the first noise exposure guidelines in 1948, further refined in 1956 to include comprehensive prevention program components that remain relevant today [11]. In 1970, the Occupational Safety and Health Act established regulations for the general industry, setting standards for safe noise exposure levels based on guidelines from the Occupational Safety and Health Administration (OSHA) and the National Institute for Occupational Safety and Health (NIOSH) [12]. OSHA has set a permissible exposure limit of 90 dBA for an 8-hour time-weighted average, with a 5-dB exchange rate in place. In 1983, an action level of 85 dBA was established, mandating the implementation of hearing conservation programs for workers at risk.

Meanwhile, NIOSH recommends an 85 dBA limit with a 3-dB exchange rate, emphasizing the necessity for a robust hearing loss prevention program for those exposed to hazardous noise levels. The ISO establishes guidelines for permissible noise levels to ensure safety, with a primary focus on steady-state exposure. Although these standards offer baseline risk assessments for noise-induced hearing loss, recent recommendations from NIOSH suggest a sound pressure level (SPL) limit of 140 dB for impulsive noises, in addition to the previously established 85 dBA for complex noise situations [13]. Emerging studies suggest that incorporating a kurtosis correction term with SPL may enhance the assessment of ONIHL risk, paving the way for more effective prevention strategies in the workplace [13, 14].

## Epidemiology

The global landscape of ONIHL reveals significant disparities in morbidity rates across different nations. The criteria for diagnosing ONIHL among workers differ widely, with the burden of occupational noise ranging from 20.4% in Kuwait to a staggering 58.5% in Tanzania (Table 1) [15–29]. Notably, regions with less economic development tend to exhibit higher prevalence rates of ONIHL, highlighting a pressing public health concern.

**Table 1:** A summary of the prevalence of noise-induced hearing loss among workers in the world by year reported by occupation by country.

Year of reported	Countries/regions	Population/exposure/sex/ age	Prevalence (hearing threshold)	Reference
2015	Nepal	Carpenters/male (N = 88) Median age: 23 years (20–31 years) Sawyers/male (N = 36) Median age: 30 years (20–45)	31% (≧50 dB) 44% (≧50 dB)	Robinson et al. [18]
2015	Tanzania	Miners workers (N = 246; Male: 243, Female: 3) Median age: 38.8 years (20–50 years)	47% (≧25 dB)	Musiba et al. [19]
2016	US	Mining workers (N = 7398; Male: 7895, Female:3) Median age: 38.7 years (18–75) Oil and gas extraction (N = 1072; Male: 977, Female: 95) Median age: 36.0 years (16–79)	24% (≧40 dB) 14% (≧40 dB)	Lawson et al. [22]
2017	Canada	Machinery or transportation workers (N = 454; Male: 352, Female: 102) Median age: 48 years (16–79 years)	37% (≧25 dB)	Feder et al. [20]
2018	Jordan	Plant male workers (N = 196) Mean age: 35.9 years (±7.2)	28.6% (≧25 dB)	Almaayeh et al. [15]
2018	US	AFFH sector (N = 17 290; Male: 12482, Female: 4808) Media age: 34.9 years (18–75)	15.0% (≧40 dB)	Masterson et al. [17]
2019	Tanzania	Textile industry workers (N = 265; Male: 161, Female: 104) Mean age: 40.3 years (±12,6)	58.5% (≧25 dB)	Abraham et al. [16]
2019	Thailand	Sawmill workers (N = 699; Male: 335, Female: 364) Mean age: 33.5 years(±10.2)	22.8% (≧25 dB)	Thepaksorn et al. [21]
2019	China	Automotive manufacturing (N = 6667; Male: 6427, Female: 240)	28.8% (≧30 dB)	Chen et al. [23]
2020	China	Transportation, mining, manufacturing industries (N = 71 865)	21.3% (≧25 dB)	Zhou et al. [25]
2021	Kuwait	Migrant workers (N = 3474)	20.4% (≧25 dB)	Buqammaz et al. [24]
2023	Egypt	Steel workers (N = 606; all workers were male)	47% (≧25 dB)	Elshaer et al. [26]
2023	Iran	Manufacturing industries (N = 85 685; all workers were male)	34.75% (≧25 dB)	Etemadinezhad et al. [27]
2024	Malaysia	Palm oil mill workers (N = 143; male: 121, female: 22)	42.7% (≧25 dB)	Mutthumanickam et al. [28]
2024	Malaysia	Marine technicians (N = 127; all were male)	29.9% (≧25 dB)	Wan Mohamed et al. [29]

Note. AFFH: agriculture, forestry, fishing, hunting.

Research consistently indicates that men are more adversely affected by occupational noise exposure than women [30]. This discrepancy may stem from men's more significant exposure in various occupational settings, influenced by differences in job roles and industries. Additionally, hormonal factors could play a role; studies suggest that estrogen may offer protective effects against hearing loss in women due to its influence on physiological pathways.

The age demographics of 30–44 and 45–59 years represent the highest risk groups for ONIHL, aligning with the peak period of labor force participation [31]. Evidence shows a decline in the affected fraction of workers over 44 years, underscoring the significant impact of occupational noise on younger individuals [1]. The longer these younger workers experience hearing loss, the more critical their contribution to the disability-adjusted life years metric becomes, signaling a longer-term burden on health systems.

Moreover, ONIHL is closely linked to an increased likelihood of job-related injuries [32]. The disruption caused by high noise levels can hinder individuals' capacity to detect essential warning signals, oversee machinery, react to ambient sounds, and communicate effectively with coworkers. Furthermore, the likelihood of hearing loss varies significantly across industries, with mining, textile, construction, and wood product manufacturing identified as the most vulnerable to ONIHL [1]. This finding underscores the necessity for targeted intervention strategies to protect workers in high-risk environments.

Long-term exposure to loud noise can have several detrimental effects on cognitive function. Here are some key impacts: [25] (i) Cognitive Decline: prolonged exposure to high noise levels has been associated with declining cognitive abilities, particularly in memory, attention, and problem-solving skills. Studies suggest chronic noise exposure can lead to difficulties in learning and retaining information; (ii) Impaired Concentration: loud environments can make focusing challenging, decreasing productivity and efficiency. Individuals may find it harder to concentrate on tasks, particularly those requiring deep thought or sustained attention; (iii) Increased Stress and Anxiety: chronic noise exposure can elevate stress levels, which may negatively impact cognitive processes. Stress hormones like cortisol can affect brain function, impairing memory and decision-making abilities; (iv) Sleep Disturbances: loud noise can disrupt sleep patterns, leading to insufficient rest. Poor sleep quality is linked to cognitive impairments, including reduced alertness, impaired memory consolidation, and decreased problem-solving skills; (v) Hearing Loss and Communication Difficulties: long-term exposure to loud noise can lead to hearing loss, which can hinder communication. Challenges in understanding conversations can result in social isolation and further cognitive decline due to reduced social interaction; (vi) Increased Risk of Neurodegenerative Conditions: previous studies suggest that chronic noise exposure might be linked to an increased risk of neurodegenerative diseases, such as dementia. The mechanisms behind this association are still being researched, but chronic stress and inflammation may play a role; (vii) Impact on Children: exposure to loud noise can significantly interfere with learning and language development in children. It can lead to difficulties in reading and academic performance, affecting long-term educational outcomes.

In summary, ONIHL is a widespread health issue among workers. Both hereditary and environmental factors play significant roles in the occurrence of ONIHL. Moreover, several demographic and health-related factors, such as age [33], pre-existing sensorineural hearing impairment, chronic conditions (e.g. diabetes, hypertension) [34, 35], a history of smoking [36], and ototoxic medications [37], can play a role in inner ear damage caused by exposure to loud noise. Extended exposure to elevated noise levels can lead to hearing impairments and substantially hinder cognitive function, creating challenges in both personal and professional contexts. Addressing noise pollution and cultivating quieter workplace environments are essential for safeguarding employee health.

## Pathogenesis

The human auditory system consists of the outer ear, middle ear, and inner ear. The inner ear includes both the vestibular system and the cochlear system. Inside the cochlea is the organ of Corti, a specialized sensory structure composed of thousands of fragile hair cells and supporting cells [38, 39]. This organ consists of two types of hair cells: inner hair cells (IHCs), which are mainly associated with most auditory nerve fibers, and outer hair cells (OHCs), which amplify mechanical stimuli and predominantly connect to efferent nerve innervations [40]. The primary role of IHCs is to transmit auditory information through multiple ribbon synapses, allowing for rapid communication without experiencing fatigue. OHCs amplify sound-induced vibrations through their contraction, which is controlled by fluctuations in membrane potential and depends on a protein sensitive to sound.

The outer ear gathers sound waves and channels them through the ear canal to the tympanic membrane, which relays sound wave vibrations to the inner ear via three tiny bones (malleus, incus, and stapes) in the middle ear [38]. These sound wave vibrations then reach the cochlea, generating an impulse that causes the basilar membrane to vibrate, allowing hair cells in the organ of Corti to transform these signals into action potentials. Effective mechanical coupling is crucial for transmitting sound energy from the air to the inner ear, specifically the cochlea. Research indicates that hair cells respond to sound through the delicate movements of their stereocilia, which contain mechanotransducer channels [40]. These channels open in response to tension from extracellular tip links connecting adjacent stereocilia, allowing for a rapid response to minor displacements, aided by calcium-dependent adaptation processes.

Additionally, the cochlea acts as a spectrum analyzer by differentiating various sound frequencies along its length, with each hair cell in the organ of Corti being specifically responsive to a specific range of frequencies. This amplification enhances frequency resolution and significantly boosts sensitivity near each hair cell's specific characteristic frequency. Genetic differences and environmental influences, such as prolonged exposure to loud noise, can lead to permanent damage to hair cells or degradation of the inner ear system. The hair cells located in the organ of Corti are susceptible to several harmful influences, including aging, high sound levels, ototoxic agents, and certain medications. Notably, exposure to high noise levels is a leading cause of permanent sensorineural hearing loss, particularly impacting OHCs [41, 42].

The effects of noise exposure on hearing loss show significant variability, shaped by the interaction of complex genetic and environmental factors. ONIHL mainly results from degenerative changes in sensory hair cells, synaptic connections, and spiral ganglion neurons within the cochlea [42, 43]. Injury to the auditory system can arise from various mechanisms, including mechanical, ischemic, synaptic, or metabolic pathways.

### Mechanical damage

Overstimulation by loud noise can cause TTS or PTS [39, 40]. Prolonged stimulation to elevated sound levels can disrupt the brain's ability to process low-frequency and high-frequency sounds [38]. Overstimulation can cause the stereocilia of hair cells to break, fuse, or detach from the tectorial membrane, while increased sound intensity can further damage hair cells and supporting structures. Severe exposure may lead to detachment between the organ of Corti and the basilar membrane, as well as the separation of endolymph from perilymph [38, 39]. While mechanical injury was once thought to be the primary cause of all NIHL, it is now understood that direct mechanical damage requires exposure to sound levels of at least 130 dB. Short bursts of loud noise can damage cochlear hair cells and the nearby supporting cells, affecting the auditory nerve fibers [38]. The level of hearing loss correlates with the intensity and duration of noise exposure, with sublethal inner ear cell injuries potentially accelerating age-related hearing loss.

### Ischemic processes

NIHL is often associated with ischemic or metabolic processes. Overstimulation of loud noise can cause vasoconstriction in cochlear blood vessels and swelling of the stria vascularis, reducing blood flow to the inner ear [39]. Overexposure to noise induces diminished blood supply. It can alter the function of hair cells and supporting structures, resulting in fused or lost stereociliary bundles after significant noise exposure, and potentially leading to the loss of nerve fibers innervating hair cells. Disorders in the stria vascularis can cause endo-cochlear dysfunction, disrupting the auditory signals amplified by the cochlea and raising auditory thresholds. Over time, this disorder may result in the death of intermediate cells, leading to a permanent reduction in blood supply to the cochlea [39].

Furthermore, the degeneration of cochlear nerve fibers occurs in conjunction with the degeneration of the central nervous system [38]. Once hair cells in mammals are damaged, they do not regenerate, making noise-induced hearing loss a permanent condition. However, some research suggests that alterations in the height of outer hair cells and the crumpling of pillar cells after noise exposure may be reversible, potentially accounting for temporary changes in hearing thresholds [38, 39].

### Synaptopathy

Research indicates that overstimulation by loud noise may also damage the synapses between spiral ganglion cells and inner hair cells, a phenomenon known as synaptopathy [42, 43]. This condition involves the reduction of spiral ganglion cells, which primarily connect with inner hair cells in the organ of Corti. This loss can result in functional hearing loss and reduced speech intelligibility, even when hair cells and supporting structures appear intact. Most studies on synaptopathy have been conducted in animal models, which may not reflect typical human noise exposure levels, leaving the generalizability to humans uncertain. Nonetheless, pathological studies of temporal bones have revealed age-related cochlear synaptopathy in human subjects, characterized by damage to hair cells in the organ of Corti. This hair cell damage highlights the necessity for further investigation into noise-induced synaptic damage in humans [42]. Currently, no clinical studies or imaging techniques are available to diagnose synaptopathy in living subjects directly.

### Metabolic and oxidative stress

**Figure 2:**
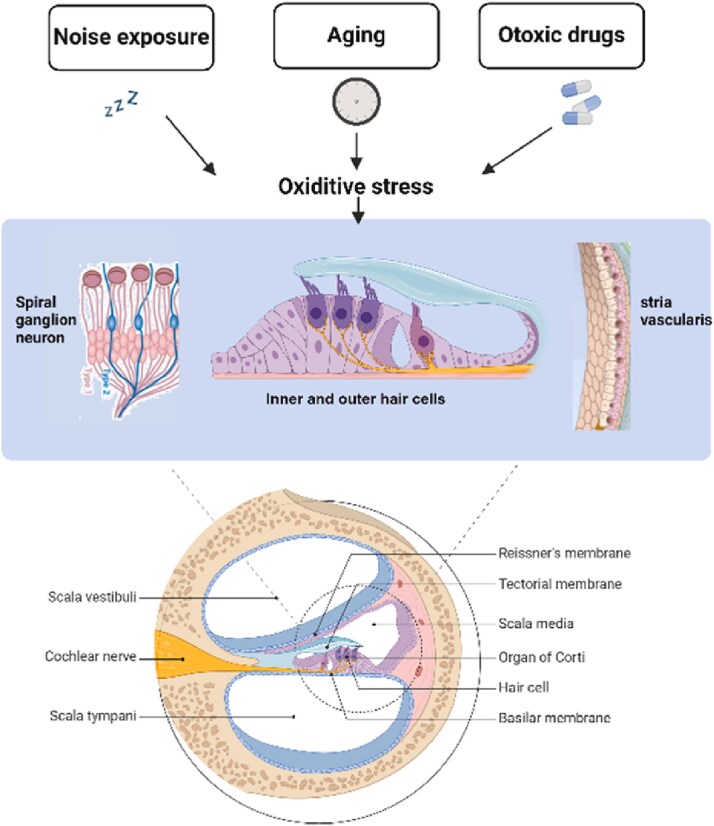
Potential pathogenesis of the cochlear related to the development of hearing loss. (photograph courtesy of Yuan C et al. [43]).

Hearing loss is a multifaceted outcome shaped by a combination of intrinsic and extrinsic factors. Aging, exposure to ototoxic drugs, and environmental noise interact to accelerate structural and functional deterioration within the inner ear. These processes manifest as degenerative changes in the stria vascularis, thickening, calcification, and hyalinization of the basement membrane, progressive atrophy of sensory hair cells, reduction in the number of supporting cells, and eventual degeneration of spiral ganglion neurons. Collectively, these pathological alterations illustrate the complex biological pathways that converge to produce NIHL, as depicted in Fig. 2 [42, 43]. Reactive oxygen species (ROS) are typical byproducts of cellular metabolism. While a certain amount is necessary for cellular functions, excessive ROS can result in oxidative damage and cell death. ROS encompasses both radical and non-radical oxygen derivatives. This broader category encompasses highly reactive oxygen molecules involved in oxidation processes, including superoxide, hydrogen peroxide, hydroxyl radicals, and singlet oxygen. Oxidative stress occurs when the balance between ROS production and the body's antioxidant defenses is disrupted, resulting in cellular dysfunction and damage [42]. The cells of the auditory system are particularly vulnerable to oxidative stress due to their high metabolic demands and are exposed to various external stressors, including overstimulation by loud noise and ototoxic medications. Research has demonstrated that exposure to noise increases ROS levels in the cochlea, activating signaling pathways that result in cell death. Hair cells, which have a limited capacity for regeneration, are especially susceptible to oxidative damage. Individuals with sensorineural hearing loss often exhibit higher levels of malondialdehyde, a marker of lipid peroxidation. Similar to those with age-related hearing loss, individuals with NIHL exhibit reduced antioxidant enzyme activity and increased oxidative stress markers. This imbalance between ROS generation and the body's ability to counteract it is critical in the etiology of hearing loss [42, 43]. The cochlea's susceptibility to oxidative stress is exacerbated by acute increases in ROS generation following noise exposure, which can persist for days and lead to necrosis and apoptosis of hair cells [42]. A recent study introduced a novel mechanism for hearing loss known as ferroptosis [44]. This process involves the dysregulation of oxidative stress and excitotoxicity, increasing ROS, intracellular iron accumulation, and lipid peroxidation. Ferroptosis is characterized by specific features, including mitochondrial shrinkage, changes in volume, and the absence of typical mitochondrial cristae. These characteristics differentiate it from classical necrosis and apoptosis, well-established forms of regulated cell death.

## Prevention and treatment

Currently, there is no effective treatment for ONIHL, making prevention the most viable strategy for addressing this condition. Key preventive measures include monitoring noise exposure, implementing engineering controls to reduce workplace noise, and ensuring regular audiometric examinations. Countries have established legal standards for occupational noise exposure, with many setting a permissible exposure limit of 85 dBA and a 3-dB exchange rate [15]. When noise reduction is not feasible, providing personal hearing protection devices (HPDs) becomes essential. Educational initiatives promoting the proper use of HPDs are critical for preventing ONIHL. Protecting your hearing in loud environments is essential to avoid noise-induced hearing loss and other auditory issues. Here are some effective strategies people commonly use [1, 15].

### Hearing protection devices

Earplugs can be placed in the ear canal to reduce noise exposure. They are available in various materials and styles, including disposable foam, custom-molded options, and reusable silicone. At the same time, earmuffs cover the entire ear and offer more significant noise reduction. They are often used in industrial settings or during activities like shooting.

### Noise-canceling headphones

These headphones utilize technology to minimize ambient noise, making them perfect for use in loud environments. They can help protect hearing while allowing for the enjoyment of music or communication.

### Limiting exposure time

Reducing time spent in loud environments is crucial. Taking breaks in quieter areas can help minimize overall noise exposure.

### Maintaining distance from noise sources

Whenever possible, staying farther away from loud machinery, speakers, or other noise sources can significantly reduce exposure levels.

### Implementing engineering controls

In workplaces, using barriers, soundproofing materials, or noise-dampening equipment can help reduce noise levels in the environment.

### Participating in hearing conservation programs

Many workplaces offer training and resources to educate employees about hearing conservation, including the proper use of hearing protection and the importance of regular hearing assessments.

### Monitoring sound levels

Utilizing sound level meters or smartphone apps can help individuals monitor noise levels in their surroundings, enabling them to make informed decisions about when to use hearing protection.

### Engaging in regular hearing check-ups

Regular hearing assessments can help detect signs of hearing loss, enabling timely intervention and improved management.

### Encouraging a culture of safety

Promoting awareness and responsibility for hearing protection among colleagues and peers can foster a safer environment, ensuring everyone takes precautions seriously.

### Interventions with pharmacological agents

To maximize the effectiveness of these devices, educational programs that emphasize the proper use of HPDs and regular hearing check-ups are essential. Furthermore, continued research into the potential benefits of antioxidants and other pharmacological agents may provide new avenues for preventing hearing loss related to oxidative stress [45]. Interventions focused on antioxidants have been introduced as promising strategies to treat and protect against NIHL, especially given the significant role of oxidative stress in the development of this condition. Research has extensively examined the potential of antioxidants to treat and protect against NIHL. These antioxidants typically comprise polyphenols such as quercetin, curcumin, resveratrol, and vitamins A, C, and E. Studies conducted on animals indicate that dietary supplements containing these antioxidants can help reduce age-related and NIHL by decreasing oxidative stress and inflammation within the cochlea. Observational studies in humans have also suggested that a higher intake of dietary antioxidants may correlate with a reduced risk of hearing loss. However, studies using randomized controlled trials are necessary to obtain evidence of the effectiveness of dietary antioxidants in preventing and treating hearing loss in humans.

Examples of these agents include N-acetylcysteine (NAC), ebselen, D-methionine, and coenzyme Q10 (CoQ10). Notably, CoQ10, recognized for its mitochondrial antioxidant properties, has been shown to diminish age-related hearing loss in both animal models and humans. Preclinical investigations have also indicated that D-methionine and ebselen may provide protective effects against hearing loss induced by ototoxic medications and noise exposure. Despite the promising nature of these pharmacological antioxidants, further clinical studies are needed to confirm their safety and effectiveness in humans. An integrated approach to preventing and managing hearing loss related to oxidative stress should encompass lifestyle modifications and strategies for noise reduction.

## Conclusion

In conclusion, ONIHL remains one of the most prevalent occupational diseases worldwide. The insights gained from this review can help policymakers identify optimal resource allocation and assess the effectiveness of previous interventions. While treatment options are still being developed, the most effective approach to reducing the incidence of ONIHL remains focused on prevention. Prioritizing preventive measures will be essential until viable treatment strategies become widely accessible.

## Data Availability

No new data were generated or analyzed for this review.
